# First molecular detection of zoonotic *Plasmodium knowlesi*, *Plasmodium cynomolgi*, and *Plasmodium inui* in Assamese macaques from northern Thailand

**DOI:** 10.1016/j.ijppaw.2025.101122

**Published:** 2025-07-25

**Authors:** Phakorn Wilaisri, Supakarn Kaewchot, Rucksak Rucksaken, Thitichai Jarudecha, Thanawat Hmaidee, Sakulchit Wichainchot, Chanapath Thabthimsri, Wanat Sricharern

**Affiliations:** a Department of Veterinary Nursing, Faculty of Veterinary Technology, Kasetsart University, Bangkok, Thailand; b Department of National Parks, Wildlife and Plant Conservation, Bangkok, Thailand; c Department of Veterinary Technology, Faculty of Veterinary Technology, Kasetsart University, Bangkok, Thailand; d Department of Parasitology, Faculty of Medicine, Kasetsart University, Bangkok, Thailand

**Keywords:** Assamese macaques, *Macaca assamensis*, *Plasmodium* spp., Thailand, Zoonotic malaria

## Abstract

The growing proximity between non-human primates (NHPs) and human communities in Thailand has raised concerns about zoonotic disease transmission. Among these NHPs, Assamese macaques (*Macaca assamensis*) are understudied in Thailand, particularly regarding their role as reservoirs for zoonotic malaria. The limited data on *Plasmodium* infections in this species highlights the need for further investigation. Therefore, this study determined the prevalence and molecular characterization of zoonotic *Plasmodium* spp. in Assamese macaques from Chiang Rai Province, northern Thailand. In total, 133 blood samples were collected from Assamese macaques at Tham Pla temple and examined using semi-nested Polymerase Chain Reaction (PCR) targeting the 18S rRNA gene. *Plasmodium* spp. DNA was detected in 32 samples (24.06 % 32/133; 95 % CI: 17.07–32.23), with nucleotide sequence analysis identifying *P. knowlesi* in 13.53 % (18/133; 95 % CI: 8.23–20.56), *P. cynomolgi* in 9.77 % (13/133; 95 % CI: 5.29–16.10), and *P. inui* in 0.75 % (1/133; 95 % CI: 0.02–4.09). To the best of our knowledge, this was the first molecular evidence of these zoonotic *Plasmodium* spp. infections in Assamese macaques in Thailand. These findings have highlighted the potential role of Assamese macaques as natural reservoirs for zoonotic *Plasmodium* species and have underscored the importance of continued surveillance. The data from this study should be beneficial in guiding future strategies to prevent and control simian malaria transmission from macaques to humans.

## Highlights

• First molecular evidence of zoonotic *Plasmodium* spp. in Assamese macaques was reported in Thailand.
• Zoonotic *Plasmodium* spp. including *P.* *knowlesi, P. cynomolgi, and P. inui* were identified using semi-nested PCR.
• *Plasmodium knowlesi* was the most frequently identified species, indicating a high risk of zoonotic transmission.
• These findings highlighted the role of Assamese macaques as potential natural reservoirs of zoonotic malaria.

## Graphical abstract

**Figure:**
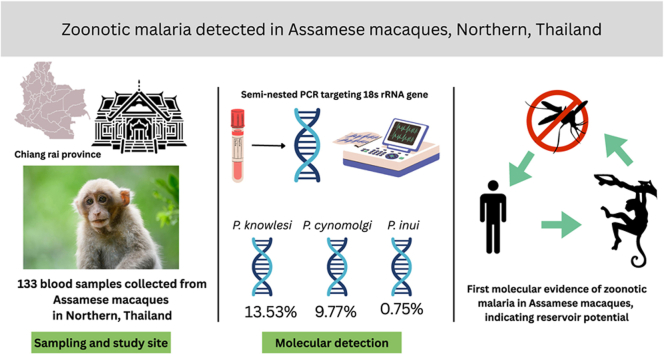

## 1 Introduction

*Plasmodium* spp.—parasitic protozoa belonging to the phylum Apicomplexa and the causative agents of malaria—continue to represent an important global public health concern (World Health Organization (WHO), 2022). The transmission cycle of malaria begins when an infected female *Anopheles* mosquito introduces sporozoites into the host's bloodstream. Subsequently, these sporozoites migrate to the liver, where they undergo asexual replication for a period of 7–10 days before developing into merozoites (Mawson, 2013; Perkins et al., 2011; Langhorne et al., 2008). Then, the merozoites are released into the bloodstream, where they invade red blood cells, resulting in clinical manifestations such as fever, anemia (Mawson, 2013). Without remedial treatment to a person bitten by some species of *Plasmodium* malaria, a life-threatening condition can develop rapidly, potentially resulting in the person's death within 24 h (Mawson, 2013; Venkatesan, 2024). To date, several *Plasmodium* species have been identified as capable of infecting humans, including *P. falciparum*, *P. vivax*, *P. malariae*, *P. ovale wallikeri*, *P. ovale curtisi* (Mawson, 2013) and zoonotic species of simian origin such as *P. knowlesi*, *P. cynomolgi* and *P. inui* which have been reported in Southeast Asian macaques (Jongwutiwes et al., 2004; Sai et al., 2022; Putaporntip et al., 2022). Despite ongoing prevention and control measures, malaria remains a major cause of morbidity and mortality worldwide (World Health Organization (WHO), 2022).

Thailand hosts a rich diversity of non-human primates (NHPs), with 14 species recorded, consisting of four species of langurs (*Trachypithecus* spp.), four species of gibbons (*Hylobates* spp.), and six species of macaques (*Macaca* spp.) (Lekagul and McMeely, 1988; Grove, 2001; Ross et al., 2014). Currently, fragmentation of natural habitats, driven by agricultural expansion and urban development, has increased the frequency of interactions between free-ranging NHPs and human communities (Seethamchai et al., 2008). Such interactions are especially frequent in villages, urban areas, and certain temples located near forests or mountainous regions (Malaivijitnond and Hamada, 2008). These temples often function as natural habitats for many NHPs and also offer food, shelter, and protection for them (Jones-Engel et al., 2006). The increasing proximity between macaques and humans in these settings raises public health concerns, especially regarding the potential transmission of zoonotic pathogens (Kaewchot et al., 2022).

In Thailand, long-tailed macaques (*Macaca fascicularis*), which are the most commonly found NHP species in the country, have been documented as hosts for various zoonotic parasitic infections (Kaewchot et al., 2022). These include *Giardia duodenalis, Cryptosporidium* spp. (Sricharern et al., 2016), *Trichuris trichiura, Hymenolepis diminuta* (Sricharern et al., 2021a), and *Bartonella quintana* (Sricharern et al., 2021b). Indeed, simian *Plasmodium* spp., which are known for their ability to infect humans, have also been detected in these macaques (Fungfuang et al., 2020).

More than 30 *Plasmodium* species have been identified in NHPs, with several of these having the capacity to be transmitted to humans, including *P. knowlesi, P. cynomolgi*, and *P. inui* in Old World monkeys (Sai et al., 2022; Fungfuang et al., 2020) as well as *P. brasilianum* and *P. simium* in New World monkeys (Lalremruata et al., 2015; Brasil et al., 2017). In Thailand, the first naturally acquired human infection with *P. knowlesi* was reported in 2004 in a patient who had visited a forested area in Prachuap Kiri Khan Province (Jongwutiwes et al., 2004). Since then, additional cases have been documented in several provinces, including Tak, Chanthaburi, Yala, Narathiwat, and Ranong (Putaporntip et al., 2009; Sermwittayawong et al., 2012; Nakaviroj et al., 2015). These reports have underscored the zoonotic risk posed by simian malaria in regions where malaria remains endemic.

**Fig. 1:**
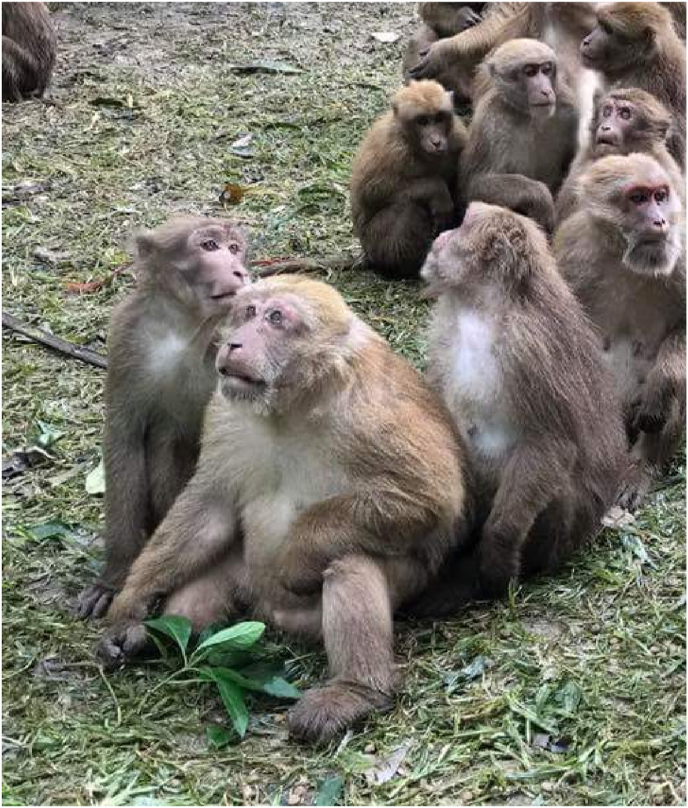
Assamese macaques at Tham Pla Temple.

Tham Pla Temple, located in Mae Sai district, Chiang Rai Province, Thailand, is one such location that attracts both domestic and international tourists. The dominant NHP species found in this area is the Assamese macaque (*Macaca assamensis*) as shown in Fig. 1 (Kaewpanus et al., 2015). This species has notable morphological variation associated with age and sex, with males generally being larger than females. The dorsal fur of this macaque ranges in color from dark brown to blackish brown, while the ventral fur varies from blond to ashy white (Boonratana et al., 2003). In Southeast Asia, including Thailand, long-tailed macaques and pig-tailed macaques (*Macaca nemestrina*) are recognized as the principal natural hosts of *P. knowlesi, P. cynomolgi*, and *P. inui* (Kaewchot et al., 2022; Fungfuang et al., 2020). However, to the best of our knowledge, there have been no molecular study detecting *Plasmodium* infection in Assamese macaques in Thailand. Therefore, the present study aimed to determine the prevalence of *Plasmodium* infection in Assamese macaques in Chiang Rai Province using molecular techniques, as well identifying the *Plasmodium* species circulating in this macaque population.

## 2 Materials and methods

This study was conducted with approval from the Animal Ethics Committee of Kasetsart University, Bangkok, Thailand (ACKU59-VTN-011).

### 2.1 Study area and sample collection

**Fig. 2:**
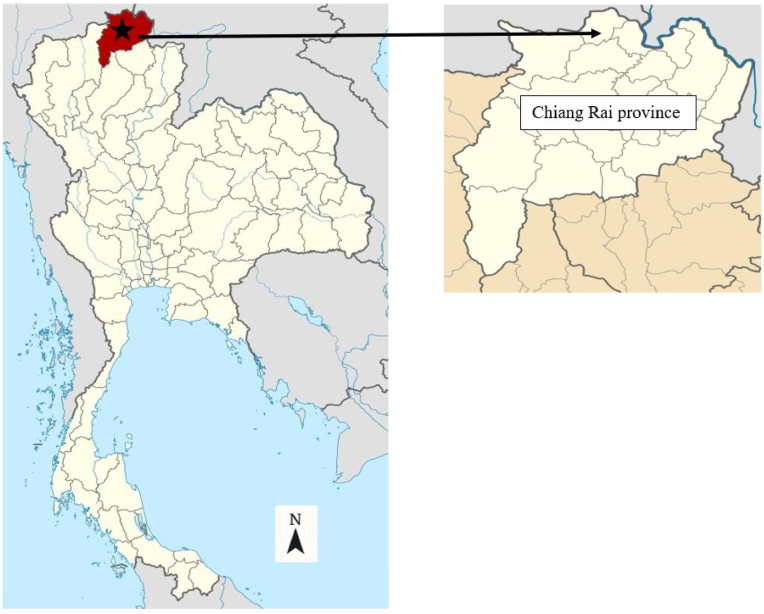
Location of sample collection site in Mae Sai District, Chiang Rai Province, Northern Thailand (Modified from https://www.pinterest.com/pin/787355947323870544/).

Blood samples from Assamese macaques were collected in July 2018 at Tham Pla Temple (Fish Cave Temple) (20°19′46.8’’N, 99°51′49.4’’E), a popular tourist destination located in the Mae Sai district of Chiang Rai province in northern Thailand (see Fig. 2). Tham Pla Temple harbors a major population of free-ranging Assamese macaques, which lived in close proximity to local residents. This proximity has facilitated frequent interactions between the macaques, tourists, and the surrounding community.

During the sample collection process, Assamese macaques were captured and anesthetized to ensure the safety of both the animals and the personnel involved. The captured macaques were initially sedated with xylazine hydrochloride (0.5–2 mg/kg body weight), followed by anesthesia with tiletamine-zolazepam (2–5 mg/kg body weight) (Fungfuang et al., 2020). Both drugs were administered via intramuscular injection, in accordance with the approved protocol. Blood was collected via venipuncture during anesthesia. After sample collection, each individual was carefully monitored until fully recovered from sedation and subsequently released back into the forest. The entire procedure was conducted under the direct supervision of veterinarians from the Department of National Parks, Wildlife, and Plant Conservation, Thailand, adhering to ethical and professional standards for wildlife handling. A total of 133 blood samples obtained from 98 male and 35 female macaques. Approximately 1 ml of blood was drawn from each individual macaque. The collected blood samples were preserved immediately in tubes containing ethylene diamine tetra-acetic acid (EDTA) as an anticoagulant. The samples were packed in ice while transported to the laboratory. Subsequently, they were stored at −40 °C for downstream DNA extraction and molecular analysis.

### 2.2 Molecular analysis

DNA was extracted from 250 μl of each of the EDTA-preserved whole blood samples using the E.Z.N.A.® Blood DNA Mini Kit (OMEGA Bio-tek Inc.; Norcross, GA, USA), following the manufacturer's instructions. The DNA concentration and quality were assessed using a Nanodrop spectrophotometer (Thermo Scientific; Waltham, MA, USA) with an absorbance ratio of 260:280 nm. The extracted DNA was stored at −40 °C until further molecular analysis.

The extracted DNA samples were analyzed using a semi-nested PCR method, adapted from Imwong et al. (2019) (Imwong et al., 2019). This method used primers targeting the 18S rRNA gene and was capable of detecting multiple species of simian malaria, including *P. cynomolgi*, *P. knowlesi*, *P. coatneyi*, *P. brasilianum*, *P. inui*, *P. simium*, *P. semiovale*, *P. fieldi*, *P. fragile*, *P. vinckei*, *P. yoelii*, *P. chabaudi*, *P. berghei*, and *P. adleria* (Karnchaisri et al., 2024).

The first round of PCR utilized the primers PlasmoM_N1F and PlasmoM_N1R, while the second round involved PlasmoM_N2F and PlasmoM_N1R. Primer sequences and thermocycling conditions are detailed in Table 1. Each PCR reaction had a 25 μl containing 1X PCR buffer, 2 mM MgCl2, 0.2 mM dNTPs, 1 μM of each primer, and 0.04 U/μl of Taq DNA polymerase (Thermo Fisher Scientific Inc., Life Technologies corporation; Carlsbad, CA, USA), with 4 μl of extracted DNA as the template. The PCR products were visualized using gel electrophoresis on a 1.2 % (w/v) agarose-Tris-acetate-EDTA (TAE) gel in 0.5X TAE buffer, run at 100 V for 30 min. Afterward, the gels were stained with GelStar® (Cambrex Bio Science; Rockland, ME, USA) and examined under ultraviolet transillumination. Positive samples with amplicons of the expected size (233–298 bp) were purified and submitted for DNA sequencing using the Sanger sequencing method (ATGC Company Limited; Pathum Thani, Thailand). The resulting sequences were analyzed using the BLAST program provided by the National Center for Biotechnology Information (NCBI; https://blast.ncbi.nlm.nih.gov/Blast.cgi) to determine sequence similarity and identify *Plasmodium* species.

**Table 1:** Oligonucleotide primers targeting 18S rRNA gene of *Plasmodium* spp., and thermal cycling conditions in the current study.

Primers	Oligonucleotide sequences (5′ ⟶ 3′)	Amplicon size (bp)	Thermal cycling conditions
Primary PCR PlasmoM_N1F PlasmoM_N1R	ATGGCCGTTTTTAGTTCGTG TTGTGTTAGACACACATCGTTCC	–	94° (5 min); × 35 at 94° for 1 min; 53° (1 min) and 72° (1 min); 72° (10 min)
Nested PCR PlasmoM_N2F PlasmoM_N1R	GTTAATTCCGATAACGAACGAGA TTGTGTTAGACACACATCGTTCC	233–298	94° (5 min); × 35 at 94° for 1 min; 53° (1 min) and 72° (1 min); 72° (10 min)

### 2.3 Statistical analysis

Statistical analyses were performed using STATA software (StataCorp; college station, TX, USA) to assess the prevalence of *Plasmodium* spp. infection in Assamese macaques, along with the corresponding 95 % confidence intervals (95 % CI). Additionally, the relationship between *Plasmodium* spp. infection and the sex of Assamese macaques was evaluated, including the calculation of odds ratio (OR) by logistic regression. The test level for significance was set at *p* < 0.05.

## 3 Results

### 3.1 Molecular detection of *Plasmodium* spp

Molecular detection using semi-nested PCR targeting the *Plasmodium* 18S rRNA gene revealed that 32 out of 133 blood samples tested positive for *Plasmodium* spp., indicating an overall prevalence of 24.06 % (95 % CI: 17.07–32.23). To determine the species involved, sequence analysis was performed, which identified only of three distinct *Plasmodium* species, with *P. knowlesi* being the most frequently detected, in 18 samples (13.53 %; 95 % CI: 8.22–20.54), followed by *P. cynomolgi* in 13 samples (9.77 %; 95 % CI: 5.31–16.13), and *P. inui* in a single sample (0.75 %; 95 % CI: 0.00–4.12).

Infection rates were compared between male and female macaques to further assess host-specific prevalence. There was a higher rate of infection in males, with 26 out of 98 testing positive, resulting in a prevalence of 26.53 % (95 % CI: 19.00–37.49). Among these, *P. knowlesi* was detected in 14 individuals (14.29 %; 95 % CI: 8.03–22.81), and *P. cynomolgi* in 12 individuals (12.24 %; 95 % CI: 6.49–20.41). In contrast, only 6 of 35 females were infected, resulting in a prevalence of 17.14 % (95 % CI: 6.58–33.65), with *P. knowlesi* found in 4 individuals (11.43 %; 95 % CI: 3.20–26.74), and both *P. cynomolgi* and *P. inui* detected in a single individual each (2.86 %; 95 % CI: 0.00–14.92). Although there was a higher prevalence of *Plasmodium* infection in males than females, the multivariable logistic regression analysis indicated no significant association between sex and infection status (OR = 0.44, 95 % CI: 0.15–1.25, P = 0.122; Table 2).

**Table 2:** Prevalence and odds ratio of *Plasmodium* infection by sex in Assamese macaques in Chiang Rai, Thailand.

Gender	No. of animals	Positive n (%)	95 % CI of prevalence	OR	95 % CI of OR	P value
Male	98	26 (26.53 %)	19.00–37.49	1 (reference)	–	–
Female	35	6 (17.14 %)	6.58–33.65	0.44	0.15–1.25	0.122
Total	133	32 (24.06 %)	17.07–32.23	–	–	–

CI: 95 % confidence intervals, OR: odds ratio.

### 3.2 Sequence analysis

Nucleotide sequence comparisons were performed using BLAST program (NCBI) to explore the genetic identity of the detected *Plasmodium* spp. Based on the results, the sequences from *P. knowlesi*-positive samples shared 98.52 % identity with *P. knowlesi* isolated from *M. fascicularis* in Malaysia (GenBank accession no. DQ350266), *Homo sapiens* from West Kalimantan, Indonesia (GenBank accession no. OR139091) and *Anopheles latens* from Sarawak, Malaysia (GenBank accession no. MN535319). Likewise, sequences from the *P. cynomolgi*-positive samples shared 98.25 % similarity to isolates from *An. balabacensis* in Sarawak, Malaysia (GenBank accession no. MN368117) and *M. fascicularis* in Malaysia (GenBank accession no. FJ619084). The *P. inui*-positive sequence shared 98.00 % identity with *P. inui* isolated from *Macaca cyclopis* in Taiwan (GenBank accession no. FN430725), *An. balabacensis* in Malaysia (GenBank accession no. MN368114) and from wild macaques in Southern, Thailand (GenBank accession no. EU400392). All of the nucleotide sequences obtained from the present study, consisting of *P. knowlesi*, *P. cynomolgi* and *P. inui* sequences, were submitted to GenBank under GenBank accession no. PQ265412–PQ625443.

## 4 Discussion

This study investigated the prevalence of *Plasmodium* spp. infections in Assamese macaques in Chiang Rai province, Thailand, using semi-nested PCR for parasite detection. Among the 133 blood samples analyzed, 32 (24.06 %) tested positive for *Plasmodium* spp. The most commonly detected species was *P. knowlesi* (13.53 %, 18/133), followed by *P. cynomolgi* (9.77 %, 13/133), and *P. inui* (0.75 %, 1/133). To the best of our knowledge, this was the first report based on molecular detection confirming natural infections with these three *Plasmodium* species in Assamese macaques in Thailand, highlighting the potential risk of simian malaria transmission in the region.

The overall infection rate observed in the present study (24.06 %) was lower than that reported in stump-tailed macaques (*M. arctoides*) in Thailand (eastern, southern, western and northeastern) where a nested PCR assay revealed a prevalence of 29.03 % (27/93) (Fungfuang et al., 2020). In that study, *P. inui* was the most prevalent species (7.53 %, 7/93), followed by *P. cynomolgi* (4.30 %, 4/93) and *P. knowlesi* (1.08 %, 1/93). For comparison, another study on long-tailed macaques across several Southeast Asian countries reported a much higher prevalence of *Plasmodium* spp. of 64.09 % (177/276), with *P. cynomolgi* being the most common (45.65 %, 126/276), followed by *P. inui* (2.90 %, 8/276), and *P. knowlesi* (0.36 %, 1/276) (Zhang et al., 2016).

However, the prevalence level in the present study was higher than that reported in another study on long-tailed macaques from Southern Thailand, where only *P. inui* was detected (6.1 %, 6/99) using a semi-nested PCR method (Seethamchai et al., 2008). The level in the present study were also higher than the prevalence recorded in wild macaques (*M. mulatta and M. fascicularis*) from 15 locations across Thailand using nested PCR, which found an overall infection rate of 13 % (100/772) (Karnchaisri et al., 2024). In that study, *P. cynomolgi* was the most frequently detected species (5.83 %, 45/772), followed by *P. inui* (4.80 %, 37/772), with no detection of *P. knowlesi*. Furthermore, free-living long-tailed macaques from various regions of Thailand had a notably lower infection rate of 2.2 % (14/649), with only *Plasmodium* spp. detected using nested PCR (Kaewchot et al., 2022). In that study, statistical analysis revealed no significant difference in *Plasmodium* infection rates between male and female Assamese macaques, consistent with previous findings in free-ranging long-tailed macaques in Thailand.

Although several *Plasmodium* species were identified in this study, all detected infections were single-species infections. However, the possibility of mixed infections in macaques cannot be ruled out. This is because the PCR assay employed was not species-specific, and identification relied on Sanger sequencing, which has limited sensitivity in detecting co-infections. This represented a limitation of the present study. Future investigations using species-specific primers and more sensitive molecular techniques are necessary to confirm the occurrence and assess the epidemiological significance of mixed *Plasmodium* infections in macaque populations.

Traditionally, *Plasmodium* infections have been diagnosed based on microscopic examination of stained blood smears, involving staining blood samples with Giemsa or Wright's stain, followed by examination under a light microscope to identify the morphological features of the parasites (Jongwutiwes et al., 2011). However, microscopic detection of *P. knowlesi* is often unreliable because the early trophozoite stages resemble those of *P. falciparum*, while the more mature, band-shaped trophozoites resemble those of *P. malariae* (Jongwutiwes et al., 2011). Several cases in Thailand and East Malaysia have identified discrepancies between microscopic and molecular diagnoses (Putaporntip et al., 2009). In some reports, infections initially diagnosed as *P. malariae* through microscopy were later identified as *P. knowlesi* through molecular analysis (Putaporntip et al., 2009; Zaw and Lin, 2019). A study conducted in Ranong, Thailand, documented two such cases, one in a Thai individual and the other in a Burmese individual. Both were initially diagnosed as *P. vivax* using microscopy; however nested PCR analysis later confirmed them as single infections with *P. knowlesi* (Sermwittayawong et al., 2012). Similarly, a case in Yala province, Thailand, involved an infection that was initially misdiagnosed as *P. vivax* based on microscopy, with subsequent molecular testing confirming a mono-infection with *P. cynomolgi* (Sai-et al., 2022). These findings highlight the limitations of traditional diagnostic techniques and emphasize the necessity of molecular methods to ensure accurate detection and species identification of *Plasmodium* infections, particularly in areas where zoonotic malaria is a growing concern.

The zoonotic potential of simian *Plasmodium* species has been documented extensively in Southeast Asia, with *P. knowlesi* being the most frequently reported species in humans. For example, high prevalence rates have been observed in Peninsular Malaysia (55.86 %, 62/111) (Vythilingam et al., 2008), Vietnam (6.05 %, 32/529) (Marchand et al., 2011), and other countries such as Singapore (Ng et al., 2008; Jeslyn et al., 2011), the Philippines (Luchaves et al., 2008), and Laos (Iwagami et al., 2018). In addition, *P. inui* has also been identified as a human-infecting parasite, particularly in Malaysia (Liew et al., 2021). In Thailand, *P. knowlesi* infections have been recorded in multiple regions, including the northwestern (Tak province), eastern (Chanthaburi province), and southern parts of the country (Yala and Narathiwat provinces) (Jongwutiwes et al., 2011), as well as in peninsular Thailand (Prachuap Khiri Khan province) (Jongwutiwes et al., 2004). In addition, *P. cynomolgi* infections have been documented in southern Thailand (Yala province) (Sai-et al., 2022).

Malaria continues to pose a major public health challenge in Thailand. According to the Ministry of Public Health, Thailand, the national malaria prevalence in 2024 was 1.64 % (14,682/896,771) (Ministry of Public Health and Thailanda). The majority of cases were caused by *Plasmodium vivax* (93.46 %, 13,722/14,682), followed by *P. falciparum* (4.62 %, 678/14,682), *P. malariae* (0.61 %, 90/14,682), *P. knowlesi* (0.53 %, 78/14,682), and *P. ovale* (0.061 %, 9/14,682). Chiang Rai Province, where Tham Pla Temple was located, reported a relatively low malaria incidence in 2024 (0.027 %, 4/14,682), and all recorded human cases were caused by *P. falciparum* and *P. vivax* (Ministry of Public Health and Thailandb). To date, no human infections with *P. knowlesi* or other simian malaria parasites had been confirmed in this region. However, the present study identified zoonotic *Plasmodium* species in Assamese macaques residing in the area. The absence of reported simian malaria in humans may have reflected underdiagnosis or species misidentification in routine surveillance.

Although Chiang Rai reported a relatively low malaria incidence in 2024 (0.027 %, 4/14,682) (Ministry of Public Health and Thailandb), the results from the present have confirmed the presence of zoonotic *Plasmodium* species in Assamese macaques in the area. Several factors may explain the relatively high prevalence of *Plasmodium* species in macaques despite the low incidence of human malaria in Chiang Rai. One possible explanation is the difference in mosquito exposure between macaques and humans, as the macaques may reside in forested or semi-natural habitats that favor contact with specific vector species. Additionally, zoonotic malaria cases in humans may be underreported or misdiagnosed due to limitations in routine diagnostic tools, especially for species such as *P. cynomolgi* or *P. inui*, which are morphologically similar to *P. vivax* (Sai-et al., 2022). Moreover, effective vector control programs and public health measures in the region may contribute to minimizing human infections.

Transmission of *Plasmodium* species between macaques and humans occurs via mosquito vectors. In Thailand, key vectors include *An. dirus*, *An. minimus*, *An. maculatus*, *An. epiroticus*, and *An. aconitus* (Ministry of Public Health and Thailandb). Studies conducted in southern, western, and northern Thailand have confirmed that these mosquito species are capable of harboring and transmitting *Plasmodium* parasites (Putaporntip et al., 2009; Sermwittayawong et al., 2012; Nakaviroj et al., 2015). However, entomological data specific to Chiang Rai province remain limited. Further research is needed to better understand the local vector populations and their roles in disease transmission in this region. It is crucial to reduce human-macaque interactions and maintain active disease surveillance to minimize the risk of zoonotic transmission. Additionally, vector control and preventive public health strategies should be prioritized to curb the spread of simian malaria.

## 5 Conclusion

The present study provided the first molecular evidence of natural infections with *P. knowlesi*, *P. cynomolgi*, and *P. inui* in Assamese macaques in Chiang Rai, Thailand, emphasizing their potential role as reservoirs for these parasites. The detection of zoonotic *Plasmodium* species underscores the public health implications, particularly in regions with frequent human-macaque contact. These findings should contribute to the growing body of knowledge on simian malaria ecology and emphasize the need for continuous surveillance, vector control, and the application of molecular diagnostics to accurately assess and mitigate zoonotic transmission risks. Further research in essential on macaque populations, vector species, and transmission dynamics to improve understanding of *Plasmodium* epidemiology and support the development of effective disease prevention strategies.

## CRediT authorship contribution statement

**Phakorn Wilaisri:** Writing – original draft, Methodology, Formal analysis, Data curation, Conceptualization. **Supakarn Kaewchot:** Methodology. **Rucksak Rucksaken:** Methodology, Funding acquisition. **Thitichai Jarudecha:** Data curation. **Thanawat Hmaidee:** Methodology. **Sakulchit Wichainchot:** Methodology. **Chanapath Thabthimsri:** Methodology. **Wanat Sricharern:** Writing – review & editing, Validation, Supervision, Project administration, Methodology, Investigation, Funding acquisition, Formal analysis, Data curation, Conceptualization.

## Declaration of generative AI and AI-assisted technologies in the writing process

During the preparation of this work the authors used ChatGPT (developed by OpenAI) in order to assist with language and grammar correction. After using this tool, the authors reviewed and edited the content as needed and take full responsibility for the content of the publication.

## Conflict of interest

There has no conflict of interest.

## References

[bib1] Boonratana R., Chalise M., Htun S., Timmins R.J. (2003). Assamese macaques (*Macaca assamensis*) in Nepal. Primate Conserv..

[bib2] Brasil P., Zalis M.G., de Pina-Costa A., Siqueira A.M., Bianco Júnior C., Silva S., Areas A.L.L., Pelajo-Machado M., de Alvarenga D.A.M., Santelli A.C.F.S., Albuquerque H.G., Cravo P., de Abreu F.V.S., Peterka C.L., Zanini G.M., Suárez Mutis M.C., Pissinatti A., Lourenço-de-Oliveira R., de Brito C.F.A., Ferreira-da-Cruz M.F., Culleton R., Daniel-Ribeiro C.T. (2017). Outbreak of human malaria caused by *Plasmodium simium* in the Atlantic Forest in Rio de Janeiro: a molecular epidemiological investigation. Lancet Global Health.

[bib3] Fungfuang W., Udom C., Tongthainan D., Kadir K.A., Singh B. (2020). Malaria parasites in macaques in Thailand: stump-tailed macaques (*Macaca arctoides*) are new natural hosts for *Plasmodium knowlesi, Plasmodium inui, Plasmodium coatneyi* and *Plasmodium fieldi*. Malar. J..

[bib4] Grove C.P. (2001).

[bib5] Imwong M., Madmanee W., Suwannasin K., Kunasol C., Peto T.J., Tripura R., von Seidlein L., Nguon C., Davoeung C., Day N.P.J., Dondorp A.M., White N.J. (2019). Asymptomatic natural human infections with the simian malaria parasites *Plasmodium cynomolgi* and *Plasmodium knowlesi*. J. Infect. Dis..

[bib6] Iwagami M., Nakatsu M., Khattignavong P., Soundala P., Lorphachan L., Keomalaphet S., Xangsayalath P., Kawai S., Hongvanthong B., Brey P.T., Kano S. (2018). “First case of human infection with *Plasmodium knowlesi* in Laos”. PLoS Neglected Trop. Dis..

[bib7] Jeslyn W.P.S., Huat T.C., Vernon L., Irene L.M.Z., Sung L.K., Jarrod L.P., Singh B., Ching N.L. (2011). Molecular epidemiological investigation of *Plasmodium knowlesi* in humans and macaques in Singapore. Vector Borne Zoonotic Dis..

[bib8] Jones-Engel L., Engel G.A., Heidrich J., Chalise M., Poudel N., Viscidi R., Barry P.A., Allan J.S., Grant R., Kyes R. (2006). Temple monkeys and health implications of Commensalism, Kathmandu, Nepal. Emerg. Infect. Dis..

[bib9] Jongwutiwes S., Putaporntip C., Iwasaki T., Sata T., Kanbara H. (2004). Acquired *Plasmodium knowlesi* malaria in human, Thailand”. Emerg. Infect. Dis..

[bib10] Jongwutiwes S., Buppan P., Kosuvin R., Seethamchai S., Pattanawong U., Sirichaisinthop J., Putaporntip C. (2011). *Plasmodium knowlesi* malaria in humans and macaques, Thailand. Emerg. Infect. Dis..

[bib11] Kaewchot S., Tangsudjai S., Sariya L., Mongkolphan C., Saechin A., Sariwongchan R., Panpeth N., Thongsahuan S., Suksai P. (2022). Zoonotic pathogens survey in free-living long-tailed macaques in Thailand. Int J Vet Sci Med.

[bib12] Kaewpanus K., Aggimarangsee N., Sitasuwan N., Wangpakapattanawong P. (2015). Behavior of Assamese macaque (*Macaca assamensis*) at Tham Pla temple, Chiang Rai province. Journal of Wildlife Thailand.

[bib13] Karnchaisri K., Day N.P.J., Dondorp A.M., Malaivijitnond S., Imwong M. (2024). Prevalence and genetic diversity of simian malaria in wild macaque populations across Thailand: implications for human health. Acta Trop..

[bib14] Lalremruata A., Magris M., Vivas-Matinez S., Koehler M., Esen M., Kempaiah P., Jeyaraj S., Perkins D.J., Mordmuller B., Metzger W.G. (2015). Natural infection of *Plasmodium brasilianum* in humans: man and monkey share quartan malaria parasites in the Venezuelan Amazon. EBioMedicine.

[bib15] Langhorne J., Ndungu F.M., Sponaas A.M., Marsh K. (2008). Immunity to malaria: more questions than answers. Nat. Immunol..

[bib16] Lekagul B., McMeely J.A. (1988).

[bib17] Liew J.W.K., Bukhari F.D.M., Jeyaprakasam N.K., Phang W.K., Vythilingam I., Lau Y.L. (2021). Natural *Plasmodium inui* infections in humans and *Anopheles cracens* mosquito, Malaysia. Emerg. Infect. Dis..

[bib18] Luchaves J., Espino F., Curameng P., Espina R., Bell D., Chiodini P., Nolder D., Sutherland C., Lee K., Singh B. (2008). Human infections with *Plasmodium knowlesi*, the Philippines. Emerg. Infect. Dis..

[bib19] Malaivijitnond S., Hamada Y. (2008). Current situation and status of long-tailed macaques (*Macaca fascicularis*) in Thailand. Nat. Hist. J. Chulalongkorn Univ..

[bib20] Marchand R.P., Culletin R., Maeno Y., Quang N.T., Nakazawa S. (2011). Co-infections of *Plasmodium knowlesi, P. falciparum, and P. vivax among* humans and *Anopheles dirus* mosquitoes, Southern Vietnam. Emerg. Infect. Dis..

[bib21] Mawson A.R. (2013). The pathogenesis of malaria: a new perspective. Pathog. Glob. Health.

[bib22] Ministry of Public Health, Thailand “Malaria situation (updated on 6 January 2024)”. https://ddc.moph.go.th/disease_detail.php?d=17.

[bib23] Ministry of Public Health, Thailand “Thailand malaria elimination program”. https://malaria.ddc.moph.go.th/malariaR10/report/malaria_doc_support_by_province.php.

[bib24] Nakaviroj S., Kobasa T., Teeranaipong P., Putaporntip C., Jongwutiwes S. (2015). An autochthonous case of severe *Plasmodium knowlesi* malaria in Thailand. Am. J. Trop. Med. Hyg..

[bib25] Ng O.T., Ooi E.E., Lee C.C., Lee P.J., Ng L.C., Wong P.S., Tu T.M., Loh J.P., Leo Y.S. (2008). “Naturally acquired human *Plasmodium knowlesi* infection, Singapore”. Emerg. Infect. Dis..

[bib26] Perkins D.J., Were T., Davenport G.C., Kempaiah P., Hittner J.B., Ong’echa J.M. (2011). Severe malarial anemia innate immunity and pathogenesis. Int. J. Biol. Sci..

[bib27] Putaporntip C., Hongsrimuang T., Seethamchai S., Kobasa T., Limkittikul K., Cui L., Jongwutiwes S. (2009). Differential prevalence of *Plasmodium* infections and cryptic *Plasmodium knowlesi* malaria in humans in Thailand. J. Infect. Dis..

[bib28] Putaporntip C., Kuamsab N., Seethamchai S., Pattanawong U., Rojrung R., Yanmanee S., Weng Cheng C.W., Jongwutiwes S. (2022). Cryptic *Plasmodium inui* and *Plasmodium fieldi* infections among symptomatic malaria patients in Thailand. Clin. Infect. Dis..

[bib29] Ross C., Boonratana R., Supriatna J., Fellowes J. (2014). An updated Taxonomy and conservation status review of Asian Primates. Asian Primates J..

[bib30] Sai-Ngam P., Pidtana K., Suida P., Poramathikul K., Lertsethtakarn P., Kuntawunginn W., Tadsaichol S., Arsanok M., Sornsakrin S., Chaisatit C., Mathavarat C., Thaloengsok S., Boonyarangka P., Thongpiam C., Demons S., Vesely B., Watres N.C., Saejeng A., Wojnarski M., Tabprasit S., Kwanpichit C., Griesenbeck J.S., Spring M. (2022). Case series of three malaria patients from Thailand infected with the simian parasite, *Plasmodium cynomolgi*. Malar. J..

[bib31] Seethamchai S., Putaporntip C., Malaivijitnond S., Cui L., Jongwutiwes S. (2008). Malaria and *Hepatocystis* species in wild macaques, Southern Thailand. Am. J. Trop. Med. Hyg..

[bib32] Sermwittayawong N., Singh B., Nishibuchi M., Sawangjaroen N., Vuddhakul V. (2012).

[bib33] Sricharern W., Inpankaew T., Keawmongkol S., Supanam J., Stich R.W., Jittapalapong S. (2016). Molecular detection and prevalence of *Giardia duodenalis* and *Cryptosporidium* spp. among long-tailed macaques (*Macaca fascicularis*) in Thailand. Infect. Genet. Evol..

[bib34] Sricharern W., Inpankaew T., Keawmongkol S., Jarudecha T., Inthong N. (2021). Molecular identification of *Trichuris trichiura* and *Hymenolepis diminuta* in long-tailed macaques (*Macaca fascicularis*) in Lopburi, Thailand. Vet. World.

[bib35] Sricharern W., Kaewchot S., Saengsawang P., Keawmongkol S., Inpankaew T. (2021). Molecular detection of *Bartonella quintana* among long-tailed macaques (*Macaca fascicularis*) in Thailand. Pathogens.

[bib36] Venkatesan V. (2024). The 2023 WHO World malaria report. Lancet Microbe.

[bib37] Vythilingam I., NoorAzian Y.M., Huat T.C., Jiram A.I., Yusri Y.M., Azahari A.H., NorParina I., NoorRain A., LokmanHakim S. (2008). *Plasmodium knowlesi* in humans, macaques and mosquitoes in Peninsular, Malaysia. Parasites Vectors.

[bib38] World Health Organization (WHO) (2022). World malaria report 2022. https://www.who.int/teams/global-malariaprogramme/reports/world-malaria-report-2022.

[bib39] Zaw M.T., Lin Z. (2019). Human *Plasmodium knowlesi* infections in south-east asian countries. J. Microbiol. Immunol. Infect..

[bib40] Zhang X., Kadir K.A., Quintanilla-Zarinan L.S., Villano J., Houghton P., Du H., Singh B., Smith D.G. (2016). Distribution and prevalence of malaria parasites among long-tailed macaques (*Macaca fascicularis*) in regional populations across Southeast Asia. Malar. J..

